# Supplementary material for: PAXX promotes KU accumulation at DNA breaks and is essential for end-joining in XLF-deficient mice
Source: Nat Commun. 2017 Jan 4;8:13816. doi: 10.1038/ncomms13816 (PMC5216128; doi:10.1038/ncomms13816)
Supplement: Supplementary Information — Supplementary Figures 1-6 and Supplementary Tables 1-2 [file ncomms13816-s1.pdf]

A.

	Weight (gram)		Count (10^6)	
	Thymus	Spleen	Thymus	Spleen
WT (n=3)	0.08±0.02	0.08±0.03	67.3±12	44.6±18
PAXX-/- (n=4)	0.07±0.03	0.09±0.02	67.7±18	37.1±10

B.

	Thymus				
x10^6	Total	DN	DP	CD4 SP	CD8 SP
WT (n=3)	67.3±12	1.8±1.2	54.2±7.0	8.7±2.2	2.5±1.2
PAXX-/- (n=4)	67.7±18	2.1±1.0	55.3±6.5	7.7±2.3	2.6±1.5

C.

	Bone Marrow			
	Pro-B%	Pre-B%	Pre/Pro Ratio	IgM+ B cells%
WT (n=3)	2.24±1.08	13.2±2.53	5.05±1.05	11.1±2.8
PAXX-/- (n=4)	2.27±1.05	14.5±3.28	6.38±1.58	10.8±3.7

D.

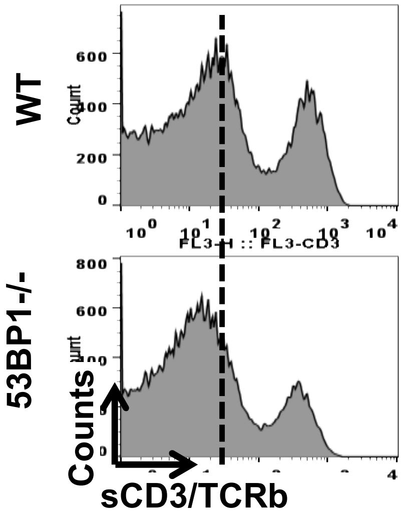

Supplementary Fig. 1 ***Paxx$^{-/-}$*** **mice have normal lymphocyte development.**

(A) The weight and total cellularity of the thymus and spleen from *Paxx$^{+/+}$* and *Paxx$^{-/-}$* mice. At least three mice at ~7 weeks of age for each genotype were analyzed and were used to calculate the average and standard derivations. (B) Total thymocyte counts and calculated $CD4^-CD8^-$double negative (DN), $CD4^+CD8^+$double positive (DP) thymocyte and single positive (SP) T cell numbers in the thymus. Each value represents the average ± standard deviation from at least three mice of ~7 weeks of age for each genotype. (C) The percentage of pro-B ($B220^+IgM^-CD43^+$), pre-B ($B220^+IgM^-CD43^-$) and mature B ($B220^+IgM^+CD43^-$) cells in total bone marrow from different genotypes. Pre/Pro Ratio was calculated from each mouse. Each value represents the average ± standard deviation from at least three mice of ~7 weeks of age for each genotype. (D) Surface CD3/TCRβ expression in thymocytes from representative *53BP1$^{+/+}$* (WT) and *53BP1$^{-/-}$* mice. The vertical line marks the median of the surface CD3/TCRβ levels in WT thymocytes.

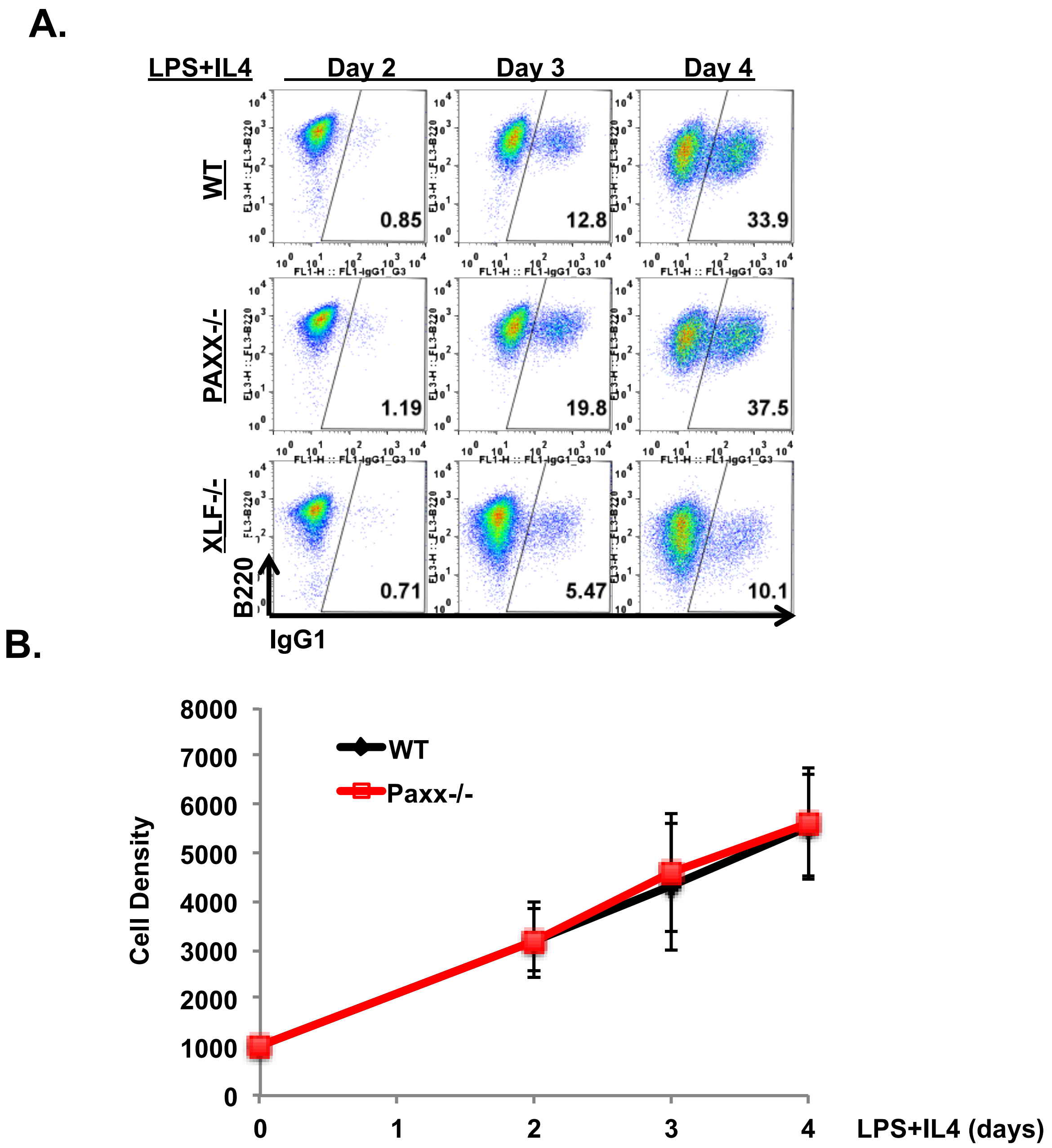

Supplementary Fig. 2 Class switching recombination is normal in *Paxx*$^{-/-}$ mice

(A) Representative flow cytometry analyses of LPS/IL4 stimulated CD43^{-} splenocytes derived from *Paxx*$^{+/+}$(WT), *Paxx*$^{-/-}$ and *Xlf*$^{-/-}$ mice at 2, 3 and 4 days after stimulation. (B) Proliferation of stimulated *Paxx*$^{+/+}$ (WT) or *Paxx*$^{-/-}$ B cells. The Y-axis indicates the cell density per µl. Cell number was counted using standard hemocytometer.

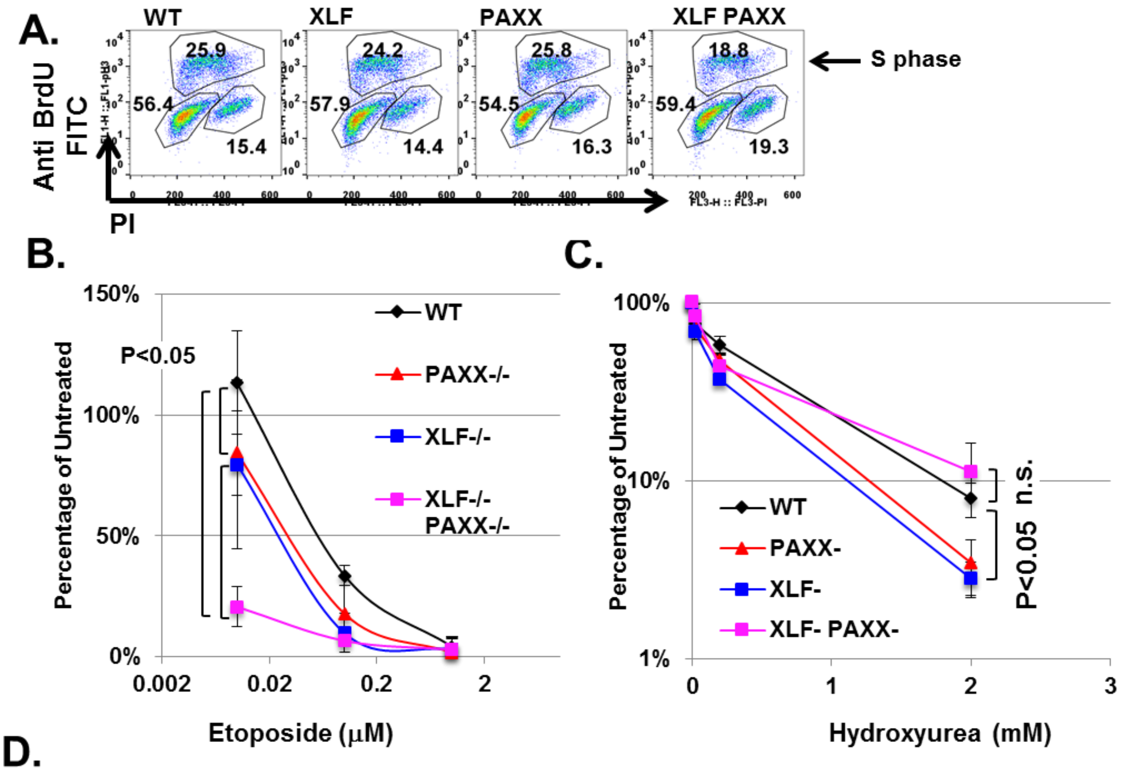

D.

	Total # MP	# abn. MP	% abn. MP	Total # abn.	Brks/ MP	Csm. Brk	Csm. Brk/MP	Ctd. Brk	Ctd. Brk/MP
**WT**	144	3	2.04%±0.13%	3	0.020	2	0.015	1	0.005
**XLF-/-**	187	18	9.63%±0.01%	18	0.096	15	0.080	3	0.016
**PAXX-/-**	144	5	3.44%±0.33%	5	0.035	4	0.028	1	0.007
**XLF-/- PAXX-/-**	206	41	18.89%±2.60%	47	0.228	42	0.196	5	0.025

E.

	Total # MP	# abn. MP	% abn. MP	Total # abn.	Brks/M P	Csm. Brk	Csm. Brk/MP	Ctd. Brk	Ctd. Brk/MP
**WT**	151	8	5.28%±0.56%	8	0.053	7	0.046	1	0.007
**XLF-/-**	150	15	10.00%±1.15%	16	0.107	13	0.087	3	0.020
**XLF-/-PAXX-/-**	169	22	13.02%±2.01%	24	0.142	18	0.137	2	0.005

Supplementary Fig. 3 Increased genomic instability in *Xlf$^{-/-}$Paxx$^{-/-}$* MEF cells and v-abl transformed pre-B cells (A) Cell cycle distribution of the indicated P1 primary MEF cells. P1 MEFs were incubated with BrdU for 30 min before collected for PI and FITC anti-BrdU staining. (B) Etoposide or (C) Hydroxyurea (HU) sensitivity assay in P1 primary MEF cells. Student's t test was used to compare the percentage of survival between different groups. n.s. not significant. The percentage (D) and (E) Summary of cytogenetic abnormalities in WT, *Xlf$^{-/-}$*, *Paxx$^{-/-}$* and *Xlf$^{-/-}$Paxx$^{-/-}$* primary MEF cells (P1) and v-abl transformed pre-B cells. Abbreviations: MP- metaphase, Abn- Abnormal, Csm-Chromosomal, Ctd-Chromatid, Brk- Break. The data summarizes the results from two or more independent experiments using at least two independently derived cell lines of each genotype. All images were obtained and processed with Metafer (Metasystem Inc).

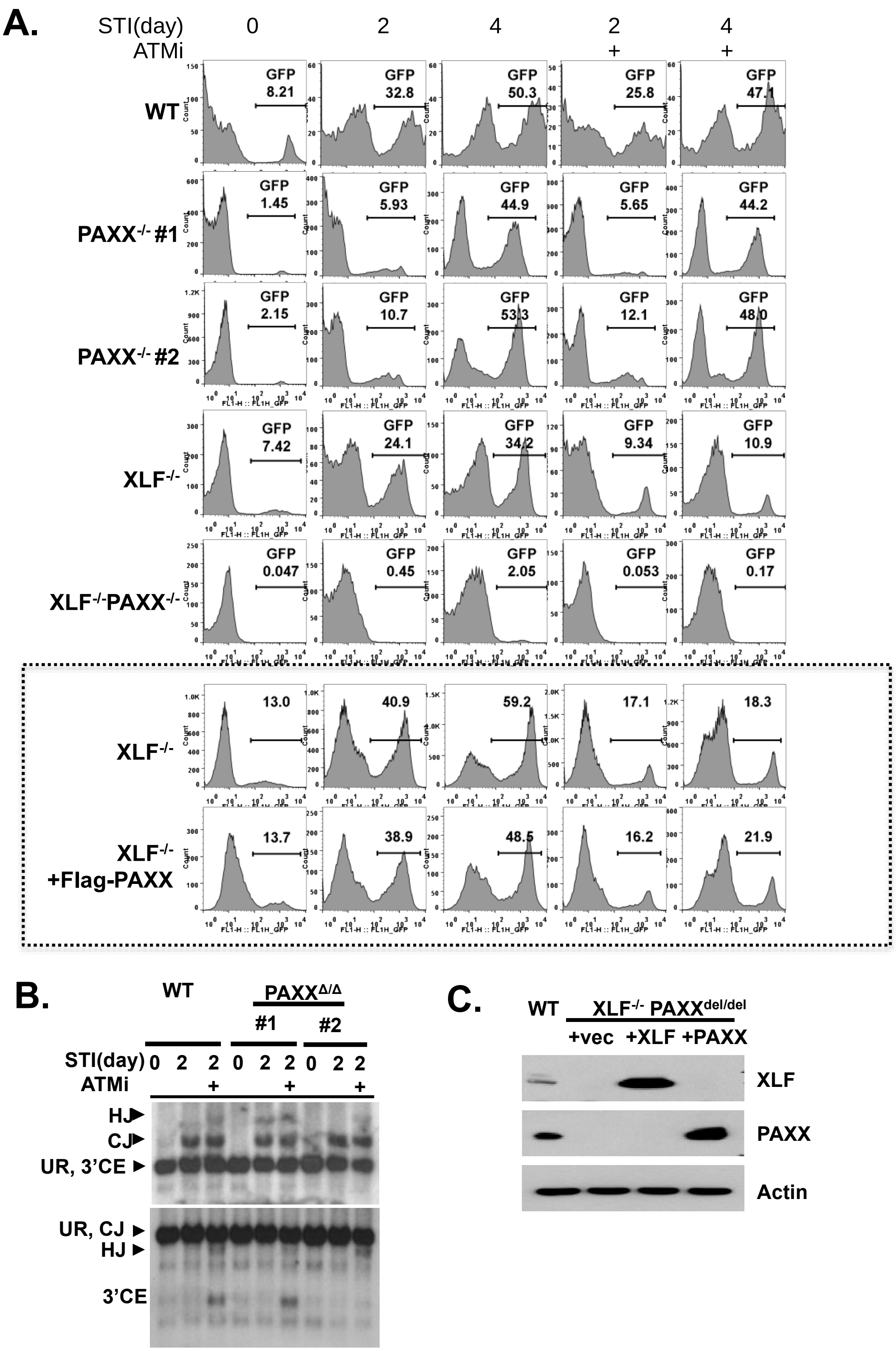

Supplementary Fig. 4 ***Xlf*** **and** ***Paxx*** **double deficiency abrogated chromosomal V(D)J recombination**
(A) Flow cytometry analyses for GFP+ cells after STI571 treatment (3µM) (with or without ATM inhibitor, KU55933, 15 µM, added 1hr before STI571) of WT, two independent $Paxx^{\Delta/\Delta}$ clones, $Xlf^{-/-}$ and $Xlf^{-/-}$ $Paxx^{\Delta/\Delta}$ cells. In parallel (lower two rows), another $Xlf^{-/-}$ cells with or without ectopic overexpression of PAXX were also treated with STI571 in the presence or the absence of ATM kinase inhibitor. Gated hCD4+ cells were plotted in the histogram. (B) Southern blot analyses of the V(D)J recombination substrates, intermediates and products of representative $Paxx^{+/+}$ (WT) and $Paxx^{\Delta/\Delta}$ cells with or without ATM kinase inhibitor (KU55933, 15 µM, added 1hr before STI571 at 3µM). See Fig.6A for the location for probes and digestion sites. (C) Western blot for XLF, Flag-PAXX and β-actin in WT and $Xlf^{-/-}$ $Paxx^{\Delta/\Delta}$ cells with or without ectopic expression of XLF or PAXX.

A.

Cell line	(Amp+Cam)^R/Amp^R	(Amp+Cam)^R/Amp^R	Relative level
Exp.1			
WT	18/15,400	0.118%	1.000
PAXX-/-	13/42,100	0.031%	0.264
XLF-/-	2/22,000	0.091%	0.078
XLF-/-PAXX-/-	1/104,500	0.001%	0.008
Exp.2			
WT	7/1,700	0.416%	1.000
PAXX-/-	33/2,900	1.149%	2.763
XLF-/-	170/20,300	0.841%	2.022
XLF-/-PAXX-/-	5/38,300	0.013%	0.032
Exp.3			
WT	82/10,600	0.777%	1.000
PAXX-/-	618/102,000	0.606%	0.779
XLF-/-	59/5,800	1.022%	1.315
XLF-/-PAXX-/-	187/149,000	0.126%	0.162
Exp.4			
WT	248/138,000	1.801%	1.000
PAXX-/-	1794/136,000	1.319%	0.733
XLF-/-	744/118,200	0.633%	0.351
XLF-/-PAXX-/-	23/142,200	0.016%	0.009

B.

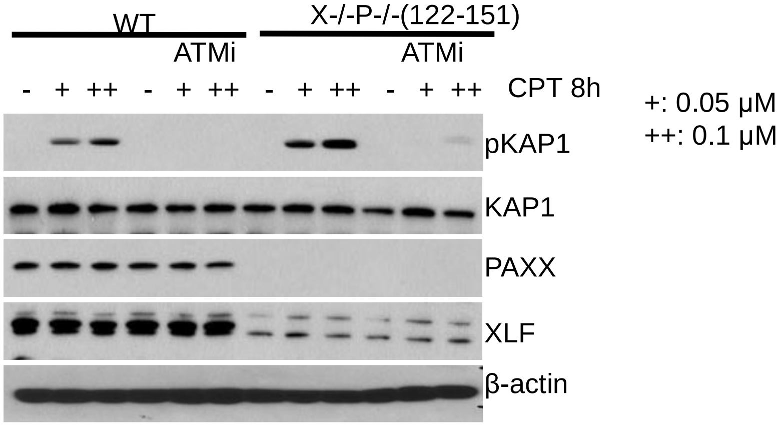

C.

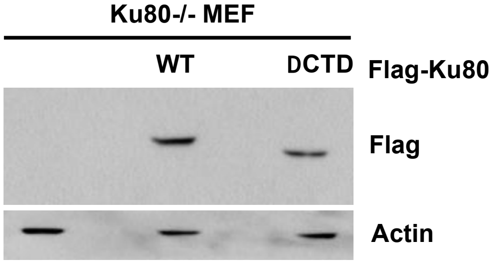

Supplementary Fig. 5 PAXX and XLF have distinct functions in NHEJ

(A) Summary of the extrachromosomal V(D)J recombination assay in WT, *Paxx*$^{\Delta/\Delta}$, *Xlf*$^{-/-}$ and *Xlf*$^{-/-}$ *Paxx*$^{\Delta/\Delta}$ v-abl transformed pre-B cells. The number and ratio of Ampicillin- Chloramphenicol double resistant (Amp+Cam)^R/ total Ampicillin resistant (Amp^R) colonies were used to calculated the relative efficiency (assumed the efficiency in the WT cells within each experiments is 1 (or 100%)). (B) Western blotting for phosphorylated KAP1, total KAP1, PAXX, XLF and β-actin in different CPT (50-100nM) treated pre-B cells. (C) The expression of full-length Flag-Ku80 and Ku80ΔCTD measured by Western blot using anti-FLAG antibody (M2, Sigma).

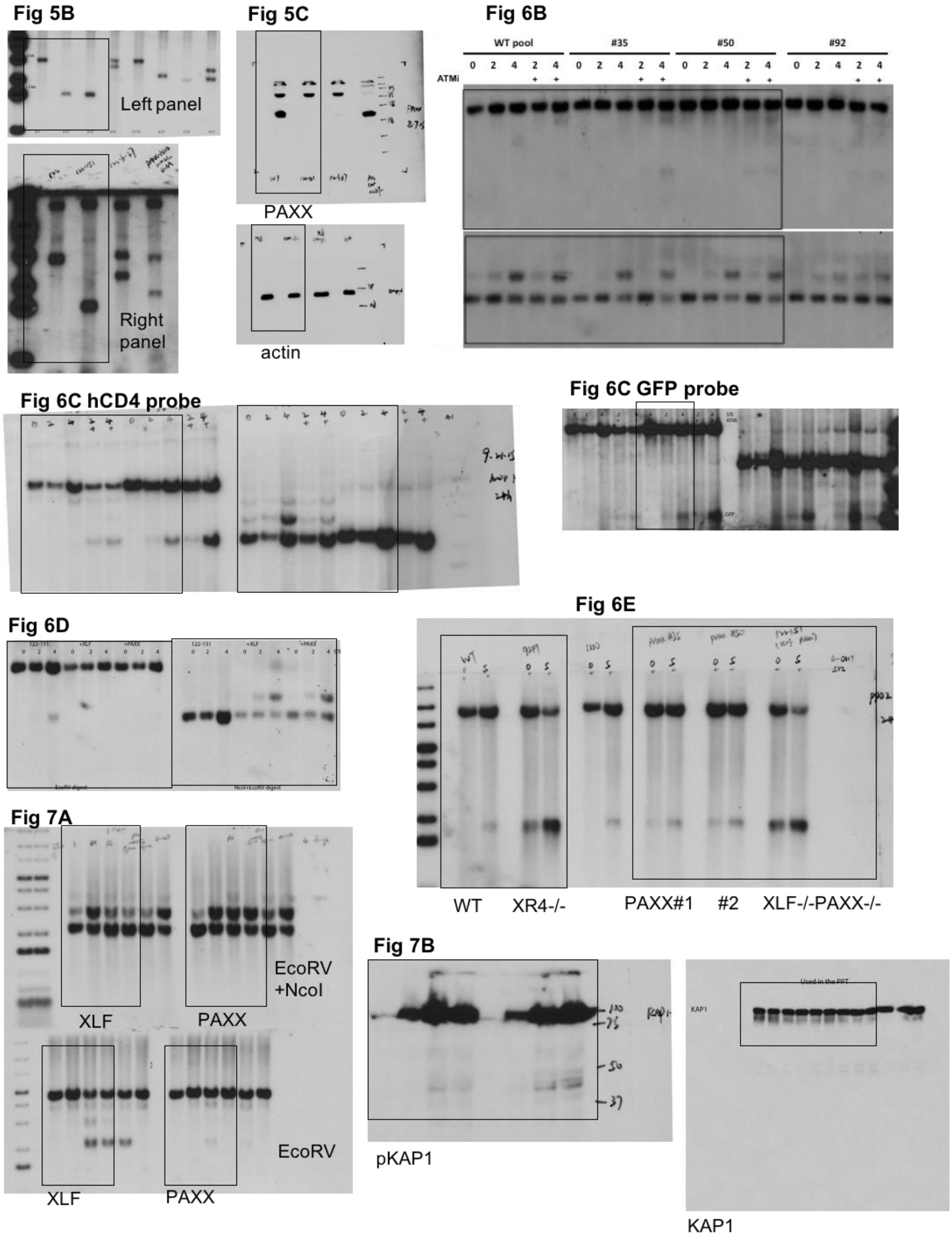

Supplementary Fig. 6 Original images of Southern blots and Western blots.

Fig 5B, 6B, 6C, 6D, 6E and 7A are original images of Southern blots hybridized with probes mentioned in paper. Fig 5C and 7B are original images of Western blots.

Supplementary Table 1 Endogenous V(D)J recombination junction at Vβ14-Dβ-Jβ1.1/2 from WT or *Paxx*$^{-/-}$ thymocytes. P: Palindromic elements, N: non-template nucleotide addition. The total number of nucleotide deletion from V-D or D-J junctions were calculated and listed on the right.

Vb14	P	N	P	Db1	P	N	P	Jb1.1 or Jb 1.2	VD del (nt)	DJ del (nt)
Paxx+/+								Jb1.1 (NTEVFFGKGTR)		
TGGCTTCTACCTCTGTGCCTGGAGTCT				GGGACAGGGGGC				CAAACACAGAAGTCTTCTTTGGTAAAGGAACCAGACT		
TGGCTTCTACCTCTGTGCCTGGAGTC			C	GGGACAGGG	C			ACACAGAAGTCTTCTTTGGTAAAGGAACCAGACT	1	6
TGGCTTCTACCTCTGTGCCTGGA				AC	G	ACGG	TG	CAAACACAGAAGTCTTCTTTGGTAAAGGAACCAGACT	7	7
TGGCTTCTACCTCTGTGCCTGGAGTCT			C	GGGGG				AAACACAGAAGTCTTCTTTGGTAAAGGAACCAGACT	6	2
TGGCTTCTACCTCTGTGCCTGGAGTCT	A	ATG		GGGGGC		A		ACAGAAGTCTTCTTTGGTAAAGGAACCAGACT	6	5
TGGCTTCTACCTCTGTGCCTGGAGTCT				AGG	C	AG		AAACACAGAAGTCTTCTTTGGTAAAGGAACCAGACT	5	5
TGGCTTCTACCTCTGTGCCT				AC	G			ACAGAAGTCTTCTTTGGTAAAGGAACCAGACT	10	12
TGGCTTCTACCTCTGTGCCTGGAGTC		A		GACAGGGG	C	GG		ACACAGAAGTCTTCTTTGGTAAAGGAACCAGACT	3	5
TGGCTTCTACCTCTGTGCCTGGAGTCT				ACAGGGG				ACACAGAAGTCTTCTTTGGTAAAGGAACCAGACT	3	5
TGGCTTCTACCTCTGTGCCTGGAGTCT		GA		GGACAGGGGG		G		ACAGAAGTCTTCTTTGGTAAAGGAACCAGACT	1	6
								Jb1.2 (NSDYTFGSGTRLLVI)		
TGGCTTCTACCTCTGTGCCTGGAGTCT				GGGACAGGGGGC				CAAACTCCGACTACACCTTCGGCTCAGGGACCAGG		
TGGCTTCTACCTCTGTGCCTGGAGTC		AGGG		AGGGGG		G		AACTCCGACTACACCTTCGGCTCAGGGACCAGG	6	3
TGGCTTCTACCTCTGTGCCTGGAG		AGACA		GGG		TGAGAG<br>G		ACTCCGACTACACCTTCGGCTCAGGGACCAGG	9	6
TGGCTTCTACCTCTGTGCCT		C		ACAGGGGGC		TCTG		ACTCCGACTACACCTTCGGCTCAGGGACCAGG	10	3
TGGCTTCTACCTCTGTGCCT	A	CCG	CC	GGGACAGGGGGC	G	GG		TCCGACTACACCTTCGGCTCAGGGACCAGG	7	5
TGGCTTCTACCTCTGTGCCTGGAGTCT				AGGGGG		GAAAT		CAAACTCCGACTACACCTTCGGCTCAGGGACCAGG	5	1
TGGCTTCTACCTCTGTGCCTGG		G	CC	GGGG				ACTCCGACTACACCTTCGGCTCAGGGACCAGG	11	5
TGGCTTCTACCTCTGTGCCTGGAG		GCGA		GGGACAGGG	C			CCGACTACACCTTCGGCTCAGGGACCAGG	3	9
TGGCTTCTACCTCTGTGCCTGGAGTCT		C		ACAGGG				AACTCCGACTACACCTTCGGCTCAGGGACCAGG	3	5
TGGCTTCTACCTCTGTGCCTGGAG	C		C	GA		G		ACTCCGACTACACCTTCGGCTCAGGGACCAGG	5	11
TGGCTTCTACCTCTGTGCCTGGAGTCT		GC		CA		CC	TG	CAAACTCCGACTACACCTTCGGCTCAGGGACCAGG	4	6
TGGCTTCTACCTCTGTGCCTGGAGTCT				AGG		AGA		AAACTCCGACTACACCTTCGGCTCAGGGACCAGG	5	5
TGGCTTCTACCTCTGTGCCTGGAGTC			C	GGACAG			TG	CAAACTCCGACTACACCTTCGGCTCAGGGACCAGG	2	5
TGGCTTCTACCTCTGTGCCTGGAGTCT	A	AA		CAGGGG		AGAAGG		AACTCCGACTACACCTTCGGCTCAGGGACCAGG	4	4
								Average	5.27	5.50
								Stdev	2.85	2.56

Supplementary Table 1 Continues

Paxx-/-								Jb1.1 (NTEVFFGKGTR)		
TGGCTTCTACCTCTGTGCCTGGAGTCT				GGGACAGGGGGC				CAAACACAGAAGTCTTCTTTGGTAAAGGAACCAGACT		
TGGCTTCTACCTCTGTGCCTGGAGTCT		CCG		GGACA		A	G	CAAACACAGAAGTCTTCTTTGGTAAAGGAACCAGACT	1	6
TGGCTTCTACCTCTGTGCCTGGAGTC			C	GACAGGGGGC	G	G		AAACACAGAAGTCTTCTTTGGTAAAGGAACCAGACT	3	1
TGGCTTCTACCTCTGTGCCTGGAG				GGGACAGGGG				CAAACACAGAAGTCTTCTTTGGTAAAGGAACCAGACT	3	2
TGGCTTCTACCTCTGTGCCTGG	C	TATGACCAATT						CAAACACAGAAGTCTTCTTTGGTAAAGGAACCAGACT	11	6
TGGCTTCTACCTCTGTGCCTGG				GGACA	T	C		CAAACACAGAAGTCTTCTTTGGTAAAGGAACCAGACT	6	6
TGGCTTCTACCTCTGTGCCTGGA		A	T	ACAGGG				AACACAGAAGTCTTCTTTGGTAAAGGAACCAGACT	7	5
TGGCTTCTACCTCTGTGCCTGGAGTC			C	GGGAC	G	G	G	CAAACACAGAAGTCTTCTTTGGTAAAGGAACCAGACT	1	7
								Jb1.2 (NSDYTFGSGTRLLVI)		
TGGCTTCTACCTCTGTGCCTGGAGTCT				GGGACAGGGGGC				CAAACTCCGACTACACCTTCGGCTCAGGGACCAGG		
TGGCTTCTACCTCTGTGCCTGGAGTCT				GGGGGC	G	G		AAACTCCGACTACACCTTCGGCTCAGGGACCAGG	6	1
TGGCTTCTACCTCTGTGCCTGG		G		GGGACAGGGGG				AAACTCCGACTACACCTTCGGCTCAGGGACCAGG	5	2
TGGCTTCTACCTCTGTGCCTGGAGTCT	AG			GGGACAGGG		AGG		ACTCCGACTACACCTTCGGCTCAGGGACCAGG	0	6
TGGCTTCTACCTCTGTGCCTGGAGTCT	A	TGG		GGGACA		A		CAAACTCCGACTACACCTTCGGCTCAGGGACCAGG	0	6
TGGCTTCTACCTCTGTGCCTGGAGTC			C	GGGACAGG	C			CTCCGACTACACCTTCGGCTCAGGGACCAGG	1	8
TGGCTTCTACCTCTGTGCCTGGAGT				GGC			TT	AAACTCCGACTACACCTTCGGCTCAGGGACCAGG	11	1
TGGCTTCTACCTCTGTGCCTGGAGTC		A		GACAG	CT	T		CAAACTCCGACTACACCTTCGGCTCAGGGACCAGG	3	5
TGGCTTCTACCTCTGTGCCTGGAGTC		CCCTAAA	C	GG				CAAACTCCGACTACACCTTCGGCTCAGGGACCAGG	1	10
TGGCTTCTACCTCTGTGCCTGGAGTC		CCGG		GGGGGC	G			CAAACTCCGACTACACCTTCGGCTCAGGGACCAGG	7	0
TGGCTTCTACCTCTGTGCCTGGAG			CC	GGGACA		A	TG	CAAACTCCGACTACACCTTCGGCTCAGGGACCAGG	3	6
TGGCTTCTACCTCTGTGCCTG		T		GGACAGGGGGG				ACTCCGACTACACCTTCGGCTCAGGGACCAGG	6	4
TGGCTTCTACCTCTGTGCCTGGAGTCT	A			GGACA		AA	G	CAAACTCCGACTACACCTTCGGCTCAGGGACCAGG	0	7
TGGCTTCTACCTCTGTGCCTGGA				CAGGGG			T	ACTCCGACTACACCTTCGGCTCAGGGACCAGG	8	5
								Average	4.15	4.70
								Stdev	3.50	2.70

Supplementary Table 2 Primers used in the study.

Primer Name	Seq 5'-3'	Note
c9orf142_DNA_H3F16758	AGAAGCTTAGAATGCCCAAGTCAGTACAC	5' ARM
c9orf142_DNA_H3R20016	CAAAGCTTAAAGCGGCAGCGACAA	5'ARM
C90rf142_DNA_F21421	CTAGGCCAGACTTGAGTTGTGA	3'ARM
C90rf142_DNA_R24826	GAAACGCAGGCCGAGATA	3'ARM
C9orf142_F24844	ACAGCAGCAGCCATCTAGG	3' probe
C9orf142_R25126	TCCAGGTTCCAAGGATATCAG	3' probe
mPAXX probe F	CACGCAAAGGCTCTAGTCC	mPAXX probe
mPAXX probe R	CGGTCTTCTTTCCTATCTATCCC	mPAXX probe
C90rf142_DNA_F19601	ATTGAAGAGCGGCAGATATGT	Genotype = KO specific (WT=2Kb, KO=0.7kb)
C90rf142_DNA_R21583	ACGCAGAATCAACACAGTAGGT	Genotype =common
C9orf142_F20809	TTGAGTGAGATGCCACGACT	Genotype =WT specific (WT=0.8Kb)
